# A new record of the spiny lobster, *Panulirusfemoristriga* (von Martens, 1872) from the coastal waters of Malaysia, with revision of global distribution

**DOI:** 10.3897/BDJ.10.e77973

**Published:** 2022-02-21

**Authors:** Wei-Ling Ng, Cheng Ann Chen, Saleem Mustafa, Chui Pin Leaw, Sing Tung Teng, Siti Nor Fatihah Binti Zakaria, Audrey Daning Tuzan, Tin-Yam Chan

**Affiliations:** 1 Borneo Marine Research Institute, Universiti Malaysia Sabah, 88400, Kota Kinabalu, Sabah, Malaysia Borneo Marine Research Institute, Universiti Malaysia Sabah, 88400 Kota Kinabalu, Sabah Malaysia; 2 Bachok Marine Research Station, Institute of Ocean and Earth Sciences, University of Malaya, 16310, Bachok, Kelantan, Malaysia Bachok Marine Research Station, Institute of Ocean and Earth Sciences, University of Malaya, 16310 Bachok, Kelantan Malaysia; 3 Faculty of Resource Science and Technology, Universiti Malaysia Sarawak, 94300, Kota Samarahan, Sarawak, Malaysia Faculty of Resource Science and Technology, Universiti Malaysia Sarawak, 94300 Kota Samarahan, Sarawak Malaysia; 4 Department of Agrotechnology and Bio-Industry, Politeknik Jeli Kelantan, Jalan Raya Timur Barat, 17600, Jeli, Kelantan, Malaysia Department of Agrotechnology and Bio-Industry, Politeknik Jeli Kelantan, Jalan Raya Timur Barat, 17600 Jeli, Kelantan Malaysia; 5 Institute of Marine Biology and Center of Excellence for the Oceans, National Taiwan Ocean University, Keelung, Taiwan Institute of Marine Biology and Center of Excellence for the Oceans, National Taiwan Ocean University Keelung Taiwan

**Keywords:** spiny lobster, first record, morphology, molecular, Sabah, global distribution, revision, checklist

## Abstract

**Background:**

Spiny lobsters of the family Palinuridae Latreille, 1802 are known to be industrial crustaceans in the global fishing market amongst other crustacean marine species. *Panulirusfemoristriga* has been reported in the Maldives, Japan, Taiwan, Vietnam, the Philippines, Indonesia (Ambon, Irian Jaya, Celebes Island, Seram Island), the Polynesian Islands, Solomon Islands, New Hebrides, Wallis and Futuna and off the coast of northern Australia, but there is uncertainty about their distributions due to the morphological similarity with *Panulirusfemoristriga*, *Panuliruslongipesbispinosus* and *Panulirusbrunneiflagellum*. However, the identification on *P.femoristiga* can only be confirmed if the morphological descriptions are mentioned in literature.

**New information:**

A specimen of the spiny lobster *Panulirusfemoristriga* Von Martens, 1872 was discovered in Semporna, located on the west coast of Sabah State, Malaysia Borneo. While the status of *P.femoristriga* has been classified as "least concern" on the International Union for Conservation of Nature Red List, studies on the species' population size, habitat and distribution are still inadequate. This study adopted both morphological and molecular approaches for species delimitation.The phylogenetic position of the Sabah *P.femoristriga* was revealed by the mitochondrial cytochrome c oxidase gene (COI) marker. This represents the first record of the species in the coastal waters of Sabah, despite its wide geographical distribution in the Indo-West Pacific. A revision on the species global distribution was also conducted by harvesting all literature with species named *Panuliruslongipesfemoristriga* and *Panulirusfemoristriga* which were available online including those prior to year 2001 before the presence of *P.femoristriga* is confirmed. Due to the uncertainties on the morphological distribution in previous literature, further studies are required to fill in the missing data for confirmation.

## Introduction

Lobsters are known to be commercially important crustaceans in the global fishing market amongst other crustacean marine species. Spiny lobsters of the family Palinuridae Latreille, 1802 are benthic, with habitats ranging in depth from shallow water to 683 metres (Booth 2001). *Panulirus* species are predominantly nocturnal and inhabit coral or rocky reefs at depths less than 40 metres (Chan 1998, Booth 2001). According to Lipcius et al. (2001), these lobsters have a home range that extends from the coastline to the continental shelf limit. They are also widely distributed and abundant in tropical and subtropical waters throughout both Hemispheres (Radhakrishnan et al. 2019). In previous research, species from the genus *Panulirus*, such as *P.polyphagus*, *P.ornatus*, *P.homarus*, *P.versicolor*, *P.penicillatus* and *P.longipes* were found in large numbers in the Indo-West Pacific (Holthuis 1991, Chow et al. 2011

*Panulirusfemoristriga* has also been found in the Maldives, Japan, Taiwan, Vietnam, the Philippines, Indonesia (Ambon, Irian Jaya, Celebes Island, Seram Island), the Polynesian Islands, Solomon Islands, New Hebrides, Wallis and Futuna and off the coast of northern Australia (Chan and Chu 1996, Juinio-Meñez and Ravago 2003, Juinio-Menez and Gotanco 2004). Despite the fact that *P.femoristriga* has been classified as "least concern" on the International Union for Conservation of Nature Red List, studies on the species' population size, habitat and distribution are scant. According to the findings of Chan and Chu (1996), the species was found in shallow rocky and coral reef environments at a depth of approximately 20 metres. However, according to a study conducted by Wahyudin et al. (2016), it was only discovered between 5 and 10 metres deep on Celebes Island and between 5 and 15 metres deep on Seram Island.

There is little information available on the distribution of marine spiny lobster (*Panulirus* spp.) in Malaysia. In Peninsular Malaysia, the primary lobster fishing grounds were on the east coast, which included East Johor, Kelantan, Pahang and Terengganu, while, in Malaysia Borneo (Sabah and Sarawak), the primary lobster fishing grounds were mostly documented on coastal islands, such as Darvel Bay (Tawau – Semporna), Tambisan Island, Banggi Island, Malawali Island, Mantanani Island and Pulau Tiga, amongst others (Biusing and Chio 2004). The species *P.polyphagus* and *P.ornatus* were both found in Johor, with the former being more abundant than the latter (Ikhwanuddin et al. 2014, Hanis and Siow 2019). Five species from Sabah waters have been reported, but only three have been identified: *P.ornatus*, *P.versicolor* and *P.longipes* (Biusing and Chio 2004). Chen and Fatihah (2018) reported *P.ornatus* and *P.versicolor* on Labuan Island. The occurrence of *P.femoristriga* in Malaysia has, therefore, not previously been reported. This study reports, for the first time, the occurrence of *P.femoristriga* in Malaysian waters (Semporna, Sabah, Malaysia), with the morphologic and molecular genetic information provided.

## Materials and methods

**Figure 1.**
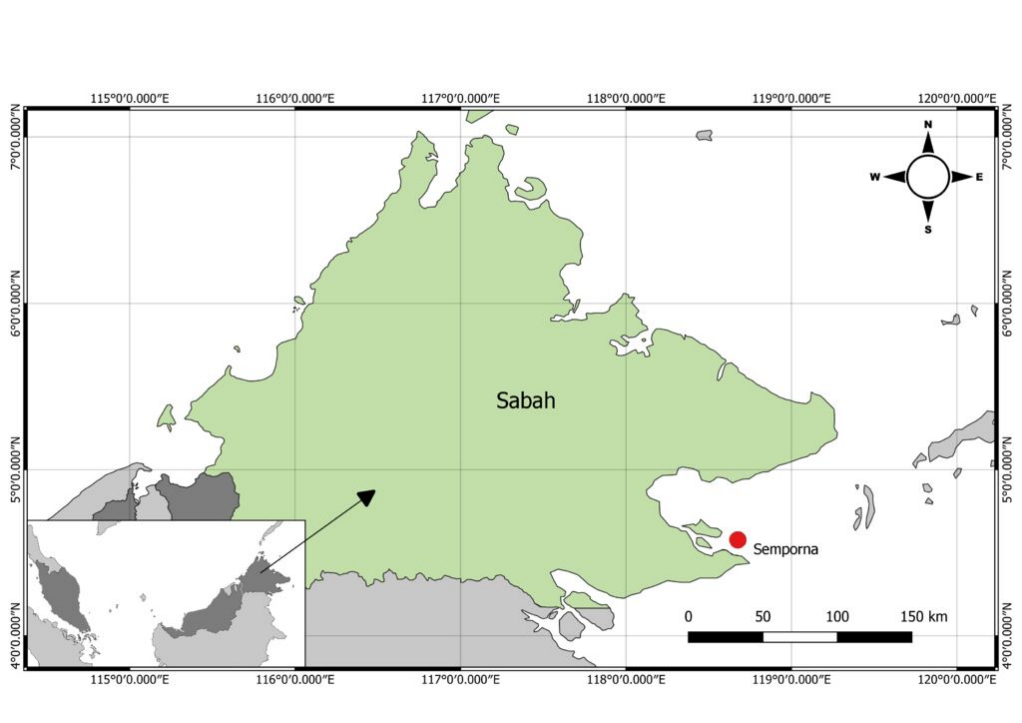
Map of Semporna, Sabah, Malaysia Borneo showing the location from which *Panulirusfemoristriga* was obtained.

The specimen was gathered from a local fisherman in November 2019. The specimen was obtained in Sabah, Malaysia, as illustrated in Fig. 1. The lobster was taken to Universiti Malaysia Sabah's laboratory for photography of the sternum, lateral view of the abdomen, antennal plate, dorsal view of the antenna peduncle, frontal horn and the entire specimen. The carapace length (CL), the carapace width (CW), the total length (TL) and the weight were measured. The morphological characteristics of lobsters were observed and the species recognised using the taxonomic keys in Chan (1998) and Chan and Ng (2001). On the other hand, due to former confusion and taxonomic issues with *P.longipes*, there is uncertainty about their distributions. Therefore, species named *Panuliruslongipesfemoristriga* and *Panulirusfemoristriga* were harvested from all literature available online, including those prior to year 2001 before the presence of *P.femoristriga* is confirmed. However, the identification on *P.femoristiga* can only be confirmed if the morphological descriptions are mentioned in literature.

For genetic identification, a small part of its abdominal tissue was dissected and fixed in ethanol for preservation. Approximately 20 mg of preserved tissue was used for total genomic DNA extraction using Vivantis GF-BT-100 Nucleic Acid Extraction kit (Vivantis, Malaysia). The mitochondrial cytochrome c oxidase subunit 1 gene (COI) was selected for phylogenetic analysis, using the universal primer pair of LCO1490 and HCO2198 (Folmer et al. 1994), LCO1490: 5'-GGT CAA CAA ATC ATA AAG ATA TTG G-3' and HCO2198: 5'-TAA ACT TCA GGG TGA CCA AAA AAT CA-3'. Gene amplification was performed as in Yamauchi et al. (2002): in a 30-µl reaction containing 18.8 µl sterile distilled H_2_O, 3 µl 10X PCR buffer, 2 µl dNTP (2.5 mM each), 2 µl each primer (5 µM), 0.2 Taq polymerase (Vivantis PL1204, Malaysia) and 2 µl DNA template (25.5 g/µl). Thirty cycles of denaturation at 98°C for 1 s, annealing at 45°C for 5 s and extension at 72°C for 10 s were used in the thermal cycle (Yamauchi et al. 2002. Electrophoresis of the amplicons was performed on a 1.0% agarose gel. The amplicons were purified using Monarch PCR & DNA Cleanup Kit prior to DNA sequencing by 1st BASE DNA Sequencing Service using Sanger Sequencing.

The nucleotide sequences obtained were used to reconstruct the phylogeny, based on Maximum Likelihood (ML). The dataset comprised of a total of 56 nucleotide sequences, including the *P.femoristriga* specimens from Malaysian waters and the closest outgroup species, *Parribacusantarcticus* Lund, 1793 (Decapoda, Scyllaridae). The outgroup was selected, based on the molecular phylogenetic investigation of Palinuridae as described in Juinio-Meñez and Ravago (2003). The programme ClustalX (Thompson et al. 2002) was used to multiple align the sequences and the Akaike Information Criterion (AIC) of jModelTest v.2.1.10 was used to evaluate and select an optimal evolutionary model (Darriba et al. 2012). The ML tree was generated using 100 bootstraps using RaxML while intra- and inter-species p-distances were determine using MEGA-X (Kumar et al. 2018).

## Data resources

- Genetic sequence has been uploaded to GenBank and can be accessed by Accession Number: OK032600
- The specimen has been deposited in Borneo Marine Research Institute collection with voucher number: IPMB-Cr 12.00001

## Taxon treatments

### Panulirus femoristriga

von Martens, 1872

9F448274-895A-5D97-B77A-53BF8F744628

OK032600

Panulirus femoristriga

#### Materials

- **Type status:** Paratype. **Occurrence:** occurrenceRemarks: purchase dead from fishermen; recordedBy: Chen CA; **Taxon:** taxonID: https://www.gbif.org/species/2221634; acceptedNameUsageID: TSN: 660231; scientificName: *Panulirusfemoristriga* (von Martens 1872); acceptedNameUsage: *Panulirusfemoristriga*; parentNameUsage: Palinuridae; originalNameUsage: *Panulirusfemoristriga* von Martens 1872; namePublishedIn: Von Martens, E. (1872). Ueber cubanische Crustaceen nach den Sammlungen Dr. J. Gundlach’s. Arch Naturgesch, 38(1), 77–147.; kingdom: Animalia; phylum: Arthropoda; class: Malacostraca; order: Decapoda; family: Palinuridae; genus: Panulirus; specificEpithet: *femoristriga*; taxonRank: species; scientificNameAuthorship: von Martens 1872; taxonomicStatus: accepted; taxonRemarks: *Panulirusalbiflagellum* Chan et Chu, 1996 (junior synonym); **Record Level:** type: collection; modified: 2001; language: en; rightsHolder: Borneo Marine Research Institute, Universiti Malaysia Sabah; accessRights: not-for-profit use only; institutionID: IPMB-Cr; collectionID: 12; datasetID: .00001; institutionCode: IPMB Crustacean collection; collectionCode: Collection locality code for Zone 5 (Lahad Datu, Kunak, Semporna, Mabul Island, Sipadan Island, Tawau); datasetName: Specimens collection number collected from the collection locality code; ownerInstitutionCode: IPMB; basisOfRecord: PreservedSpecimen

#### Description

Rostrum absent; carapace rounded and spiny; anterior margin armed with irregular-sized spines; median area behind frontal horns generally with a longitudinal row of three spines only (Fig. 3a); cervical groove distinctly wider than posterior marginal groove; antennules with flagella longer than peduncle; antennular plate at basis of antennae bearing one pair of well-separated principal spines and some scattered spinules (Fig. 3b); ventral surfaces of distal two antennal segments, each with a row of two equal-sized large spines only (Fig. 3d). First 4 pereiopods without pincers; Thoracic sternum with two strong submedian protrusions (Fig. 3c). Abdominal tergites with a complete transverse groove joining the pleural groove; pleura only with that of second somite sometimes bearing spinules (Fig. 3e). Colour: body dark brown to indigo, covered with numerous white spots and markings; lateral carapace with two longitudinal white stripes running along the entire carapace; inner surface of antennal peduncle (including stridulating pad) and antennular plate pink; antennal flagella dorsally brown, ventrally whitish to pink; antennules with peduncle dark brown but laterally white, outer flagella dark brown and inner flagella entirely whitish; legs striped with conspicuous white lines; abdomen covered with numerous medium-sized white spots. Soft part of tail fan orange-brown with distal margin whitish. Pleopods somewhat greenish with white margins.

#### Diagnosis

**Figure 2.**
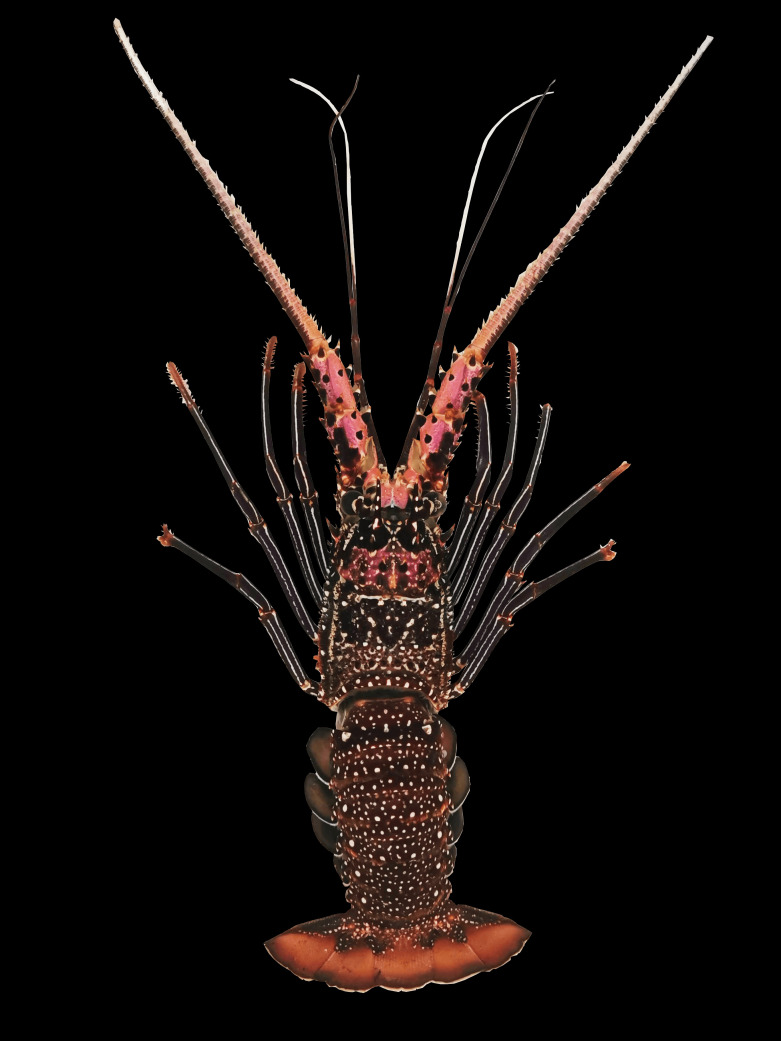
A specimen of *Panulirusfemoristriga* collected from Semporna, Sabah, Malaysia. (female CL 47.3 mm, TL 145 mm).

**Figure 3.**
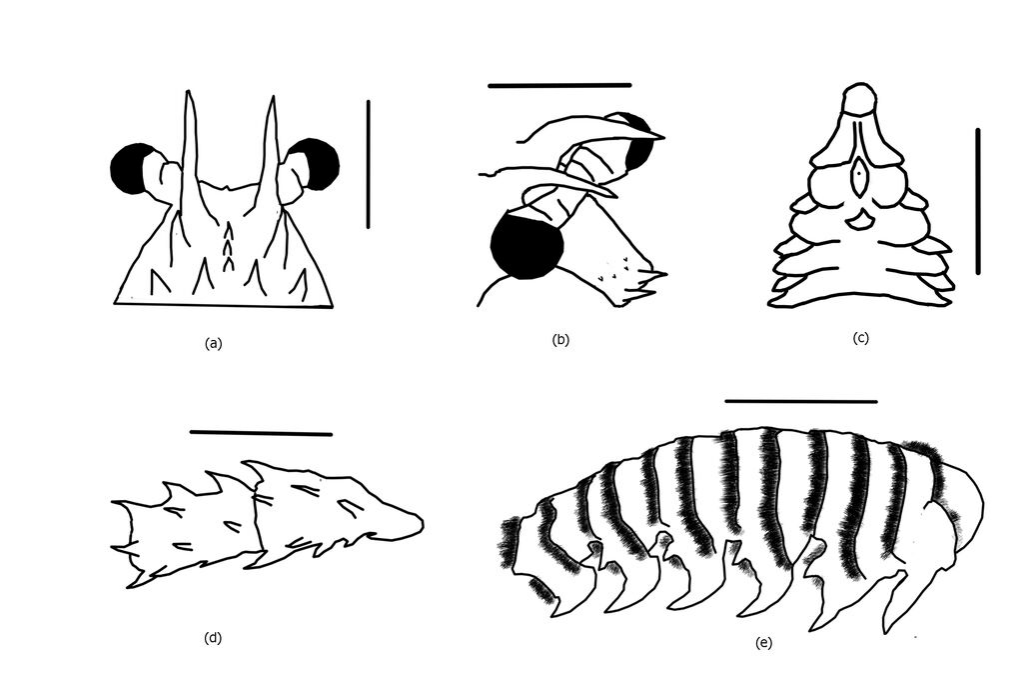
Morphological drawings: (**a**) Anterodorsal carapace, dorsal; (**b**) Antennular plate, dorsolateral; (**c**) Thoracic sternum, ventral; (**d**) Thoracic sternum, ventral; (**e**) Abdomen, right lateral. Scale bars, 2 cm.

**Examined materials.** 1 female: Malaysia; Sabah; Semporna; 8 December 2019. Carapace length 47.3 mm, carapace width 40.44 mm, total length 145 mm, weight 129 g. (Fig. 2)

##### Molecular characterization

**Figure 4.**
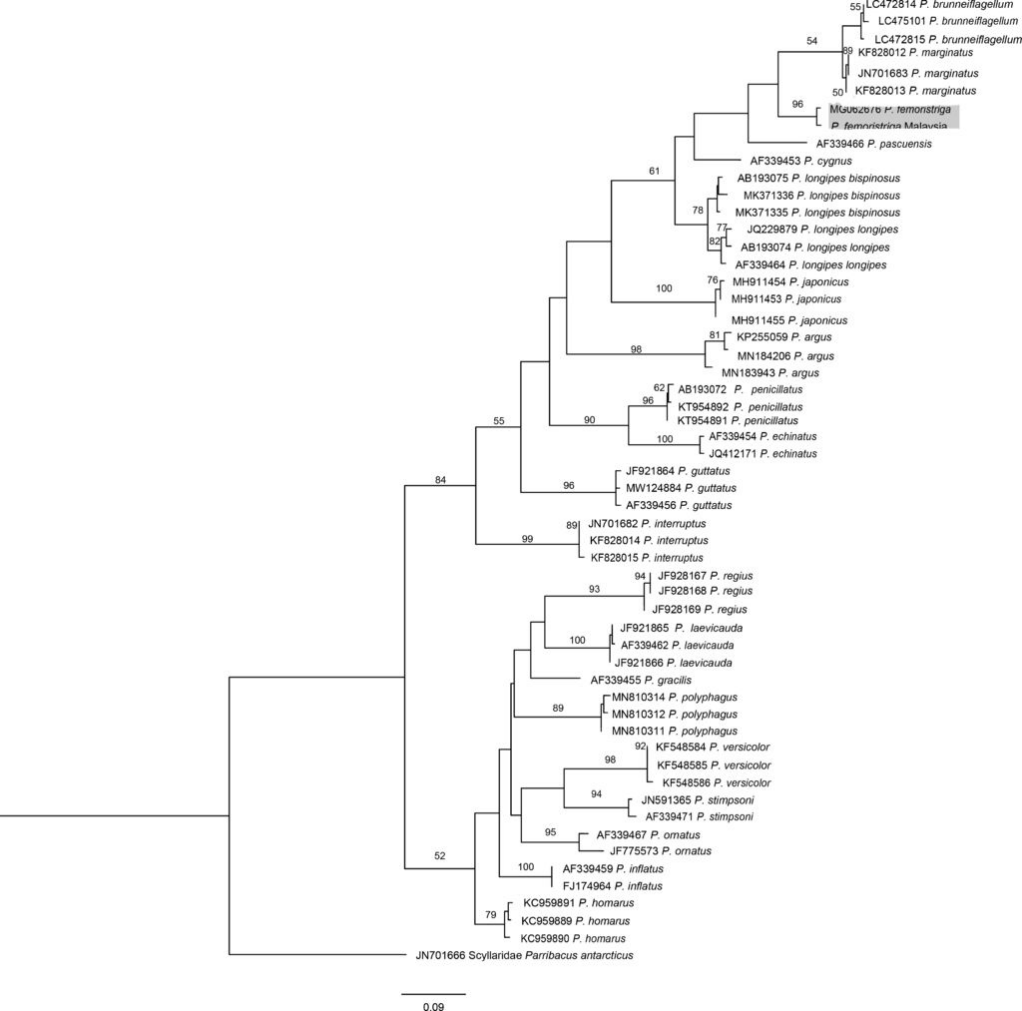
Maximum Likelihood (ML) tree inferred from the partial mitochondrial COI sequences of 56 species of *Panulirus*, with *Parribacusantarcticus* as outgroup. Values of the nodes correspond to bootstrap values, only values > 50 are shown.

The best model of the COI fragment dataset in this study estimated by jModelTest selected with corrected AIC was the GTR+I+G model (General Time Reversible Model with invariant sites and non-uniform evolutionary rates or gamma distribution). Overall, the ML tree of COI comprised two major clades (Clade I and Clade II) as shown in Fig. 4. The collected specimen in this study grouped in Clade I together with *P.femoristriga* (MG062676) from Indonesian waters, with strong bootstrap value of 96%. *Panulirusfemoristriga* grouped as sister species with *P.marginatus* + *P.bruneiflagellum*, but with weak bootstrap value (< 50%, not shown in the tree). The p-distance within *P.femoristriga* was 0.8%, while the p-distance of *P.femoristriga* with molecular (*P.marginatus* and *P.bruneiflagellum*) and morphological (*P.longipeslongipes* and *P.longipesbispinosus*) closely-related species were more than 10% (Table 1). Accordingly, the spiny lobster in this study is confirmed as *P.femoristriga*. For information and record, a new sequence dataset is uploaded to GenBank, with the accession number: OK032600.

#### Distribution

**Figure 5.**
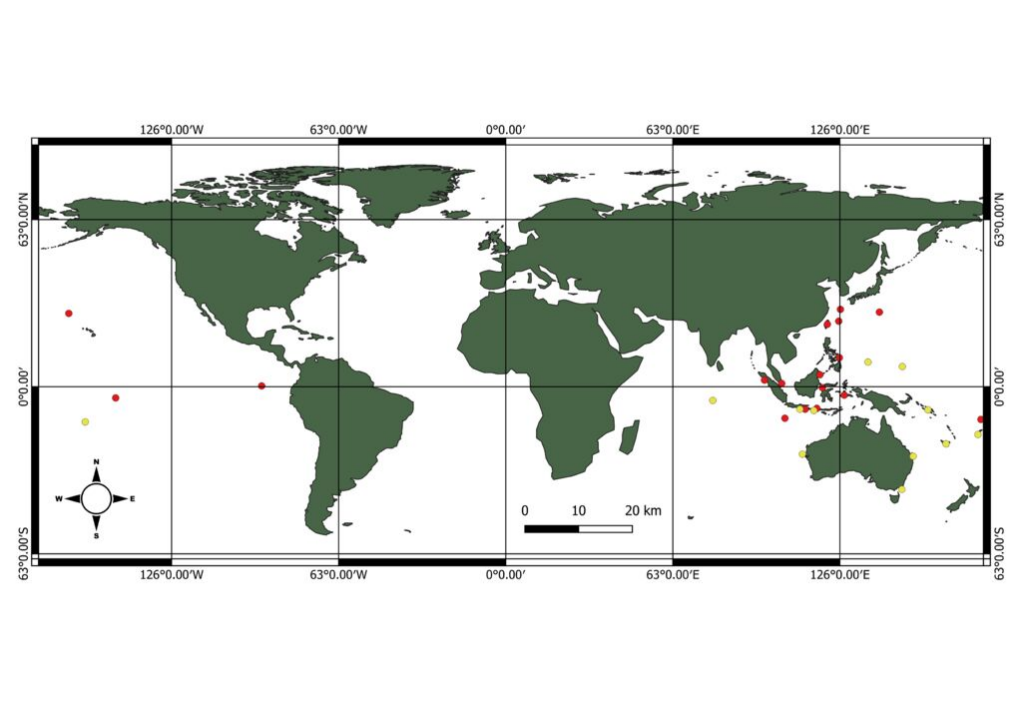
World map of *Panulirusfemoristriga* (red spot, distribution of *P.femoristriga*; yellow spot, to be confirmed)

Based on Table 2, the distributions were confirmed in Christmas Island, Wallis and Futuna Island, Marquesas, north-western Hawaiian Islands, Galapagos Islands, Taiwan, Philippine, Indonesia and Japan. Fig. 5 shows the status of *Panulirusfemoristriga* in a world map. This study expands its global distribution by including Malaysia. However, some of the distribution localities are listed as “to be confirmed” due to the uncertainties or “invalid” after the revision on the new taxonomic restructuring by Chan and Ng (2001), based on the morphological descriptions. The results indicated that the occurrence of the *P.femoristriga* needs a major re-confirmation in some localities or be removed.

## Identification Keys

### Key to the species of *Panulirus* occurring in Malaysia

**Table:** 

1	Transverse groove of second abdominal segment joining corresponding pleural groove	2
–	Abdomen without transverse grooves or only with broad sunken pubescent areas	3
2	Median area at anterior carapace behind frontal horns usually bearing a longitudinal row of 3 spines only; ventral surfaces of distal 2 antennal segments each with 2 large spines only; thoracic sternum with 2 strong submedian protrusions; antennules with outer flagella dark brown and inner flagella entirely whitish; antennal peduncle including stridulating pad pinkish; lateral carapace with 2 complete longitudinal white strips extending along the entire carapace; legs striped.	*Panulirusfemoristriga*
–	Median area at anterior carapace behind frontal horns always bearing some smaller, irregular spines in addition to the regular row of 3 spines; ventral surfaces of distal 2 antennal segments each with 1 large spine (sometimes also with several other scattered spinules); thoracic sternum without strong submedian protrusions; antennules alternated with dark brown and white bands; antennular peduncle brown to purple and with stridulating pad bright blue; lateral carapace with 1 short (upper) and 1 long (lower) longitudinal white stripes; legs striped or spotted.	*Panuliruslongipes*
3	Abdomen naked and smooth; legs blotched.	4
–	3b. At least second and third abdominal segments with broad sunken pubescent areas; legs striped.	*Panulirusversicolor*
4	Antennular plate armed with 1 pair of principal spines; body pale green and abdomen with narrow transverse yellowish-white bands.	*Panuliruspolyphagus*
–	Antennular plate armed with 2 pairs of principal spines; body greenish and abdomen with broad transverse dark bands, legs and antennules conspicuously ringed with light yellow and black.	*Panulirusornatus*

## Discussion

Molecular phylogeny is an alternative way for species identification, it avoids possible misidentification and further verifies the identity of a specimen. Species identification is particularly difficult for spiny and slipper lobster phyllosoma larvae as they are almost morphologically similar and the morphological descriptions available are not based on confident species identities. However, many studies now have successfully delimited phyllosoma larvae by employing COI barcode technique (Chow et al. 2006, Palero et al. 2014, Konishi et al. 2019, Ueda et al. 2021) and also have consistent results with the adult specimen (Govender et al. 2019). On the other hand, a study has revealed that *P.femoristriga* (formally known as *P.longipesfemoristriga*) formed sister species to *P.longipeslongipes* (Ptacek et al. 2001). Juinio-Meñez and Ravago (2003) later provided strong support to the nomenclature of the previous study as they analysed the mitochondrial DNA (mtDNA) COI sequences to distinguish the genetic variability between the ‘banded whisker’ and ‘white whisker’ of *P.longipesfemoristriga.* The result of their phylogenetic analysis not only showed the distinction of *P.femoristriga* from *P.longipesbispinosus*, but also clarified the phylogenetic relationships amongst the closely-related species, such as *P.longipeslongipes* and *P.longipesbispinosus.* The COI phylogenetic tree in this study showed that the collected specimen from Semporna is *P.femoristriga*. Based on the phylogeny, *P.femoristriga* is firstly found to group together with *P.marginatus* + *P.bruneiflagellum*. Previously, it grouped with *P.longipeslongipes* (Ptacek et al. 2001) and *P.cygnus* + *P.marginatus* + *P.longipeslongipes + P.longipesbispinosus* (Juinio-Meñez and Ravago 2003). *Panulirusfemoristriga* grouped distinctly from its morphologically closely-related *P.longipeslongipes* and *P.longipesbispinosus*, which is congruent with the phylogenetic grouping in Juinio-Meñez and Ravago (2003).

*P.femoristriga* was first described by Von Martens (1872) in Berlin, with the type locality of the specimen in Amboina, Indonesia. It was once considered as a subspecies of *P.longipes*, designated as *P.longipesfemoristriga*, due to the small- to medium-sized white spots plastering the abdomen and the pereiopods being striped instead of spotted. Chan and Chu (1996) discovered that there were two forms of *P.longipesfemoristriga* which exhibited different colouration and were genetically distinct. Presuming that the type of *P.longipesfemoristriga* was lost, a new name *P.albiflagellum* Chan and Chu, 1996 was given to the present form with the erecting of a neotype for *P.longipesfemoristriga*. Nevertheless, the holotype of *P.longipesfemoristriga* was later found and revealed that it belongs to the same species as *P.albiflagellum* (Chan and Ng 2001). *Panulirusalbiflagellum*, therefore, becomes a junior subjective synonym of *P.femoristriga*.

*P.femoristriga* is classed as "least concern" in the IUCN Red List with the last assessment date being in December 2009. Generally, *P.femoristiga* is present in the South Pacific Islands (George 1972, Sekiguchi 1997) and Indo-west Pacific (George 1965, Sekiguchi 1997). However, many of the distribution localities cannot be confirmed due to the uncertainties in morphological descriptions that could lead to misidentification between *P.femoristriga, P.l. bispinosus* and *P.brunneiflagellum* and that needed further clarification for confirmation due to the lack of information on the colour of the flagella (white and not banded on inner flagella for *P.femoristriga*) in literature. Although all species are characterised with striped-leg (Sekiguchi and George 2005), each species can be distinguished using the colour of the flagella: *P.l.bispinosus* (white-banded whisker), *P.femoristriga* (plain white whisker) and *P.brunneiflagellum* (brown whisker) (Chan and Ng 2001, Sekiguchi and George 2005). Therefore, previous records without this information is in a dilematic situation to conclude the actual species that presence in some localities (refer to Table 2 on “To be confirmed”). Furthermore, species in the study of Pitcher (1992) are unable to be determined as they not only lack morphological description, but, based on the statement in the text, Nei's distance showed subspecies status to *P.longipes.* Yet, the results on the genetic profile and Nei's distance is missing from the report and the original reference (Macaranas et al. unpublished data), which was widely cited, is not available online (invalid since it was only mentioned when submitted to the Journal of Crustacean Biology, but never published).

The recent finding enriches Malaysian seas' marine biodiversity lists. The latest and most complete record on the distribution of *Panulirus* spp. in Malaysia was by Biusing and Chio (2004). Subsequently, only limited research (Ikhwanuddin et al. 2014, Chen and Fatihah 2018, Hanis and Siow 2019) has been conducted regarding the distribution of the spiny lobster, but information was very limited. Nonetheless, *P.longipes* is a common species in Malaysia and the fishermen may consider them in general as red lobster (local term ‘udang karang merah’). Local fishermen usually categorise these lobsters according to their colour, for instance, *P.ornatus* as blue lobster and *P.versicolor* as green lobster. Therefore, it is possible that *P.femoristriga* has been mistaken for *P.longipes* due to the very similar morphology, such as colouration and the white spot on their abdomen. However, further study is required to determine whether the species was often being mistaken or that it is rare in Malaysia.

Further information about marine biodiversity is strongly linked to the resolve to protect the natural riches of the globe, particularly marine life. Considering that spiny lobsters are target fish industry species, it is crucial for its sustainable fisheries management to acquire biological knowledge. As we are aware, 17 sustainable development goals were established and launched in 2015 by the United Nations, with 169 targets for the next 15 years. In the perspective of marine diversity and sustainable development, the 14th objective (life under water) of the agenda is to preserve and sustainably utilise the oceans, seas and marine resources by the marine system. This study would also be utilised to promote sustainable resource conservation planning in the research study and to enhance the region's ecological function. Biological knowledge on *P.femoristriga* is required in future to discover approaches to ensure its sustainable utilisation and management.

## Supplementary Material

XML Treatment for Panulirus femoristriga

## Figures and Tables

**Table 1.** Pairwise p-distance of *Panulirusfemoristriga* and its closely related species. N, number of sequences.

	*P.femoristriga*	*P.longipes*	*P.bruneiflagellum*	*P.marginatus*	*P.bispinosus*
*P.femoristriga*	0.0088	0.1482-0.1593	0.1283-0.1372	0.1283-0.1327	0.1504-0.1615
N	2	6	6	6	6

**Table 2.** Checklist of *P.femoristriga*.

Country	Location	Status	Reference
Australia	Christmas Island	Present (Previously identified as *P.longipes*)	George (1968), Ng and Naruse (2014)
	Western Australia/ East Indian Ocean	To be confirmed (Lack morphological description on *P.l.femoristriga*)	Chittleborough and Thomas (1969)
	South-eastern Australia	To be confirmed (Lack morphological description on *P.l.femoristriga*)	McWilliam and Phillips (1983)
	Eastern Australia	To be confirmed (Lack morphological description on *P.l.femoristriga*)	Dennis et al. (2001)
France	New Caledonia	To be confirmed (Reference used not valid)	Pitcher (1992)
		To be confirmed (*P.l.femoristriga* in text, but morphology description resembles *P.l.bispinopsus* -Translation from French)	Richer de Forges and Laboute (1995)
		To be confirmed (Lack morphological description on *P.l.femoristriga*)	Coutures (2000)
	Wallis and Futuna Island	Present	Poupin and Junker (2008)
French Polynesia	Marquesas	Present	Curtiss (1938)
	Tahiti	Most likely belong to *P.l.bispinosus*	Curtiss (1938), Ng et al. (2011)
Fiji	Fiji Islands	To be confirmed (Reference used not valid)	Pitcher (1992)
Micronesia	Micronesia	To be confirmed (Reference used not valid)	Pitcher (1992)
Solomon Islands	Solomon Islands	To be confirmed (Reference used not valid)	Pitcher (1992)
		To be confirmed (Lack morphological description on *P.l.femoristriga*)	Richards et al. (1994)
United States	Palau	To be confirmed (Reference used not valid)	Pitcher (1992)
	North-western Hawaiian Islands	Present	O'Malley and Walsh (2013)
Ecuador	Northern shore of Isabela, Galapagos Islands	Present (Previously identified as *P.albiflagellum*)	Bustamante et al. (2002)
Taiwan	Taiwan	Present; (Previously identified as *P.albiflagellum*)	Chan and Chu (1996)
Philippine	Guiuan (Eastern Samar)	Present	Juinio-Meñez and Ravago (2003)
Singapore	Singapore	Present	Ptacek et al. (2001), Yeo et al. (2011)
Indonesia	Amboina	Present	Von Martens (1872), Holthuis (1991), Chan and Chu (1996), Chan and Ng (2001), Ptacek et al. (2001)
	Watukarung, Pacitan	To be confirmed (Lack morphological description on *P.l.femoristriga*)	Bakhtiar and Sartiyatno (2014)
	Lombok Island	Present	Kalih (2012)
	Celebes Island	Present	Wahyudin et al. (2016)
	Sepi Bay	To be confirmed (Lack morphological description on *P.l.femoristriga*)	Nurfiarini and Purnamaningtyas (2017)
	Seram Island	Present	Wahyudin et al. (2017)
	Gulf of Prigi	Present	Wijaya et al. (2017)
Japan	Amami-Oshima Island	Invalid; it belongs to *P.l.bispinopsus* based on description	Matsuda and Yamakawa (2000)
	Ogasawara Islands	Present	Sekiguchi (1997), Sekiguchi and Inoue (2002), Sekiguchi and George (2005)
	Ryukyu Archipelago	Present	Sekiguchi (1997), Chow et al. (2006), Fujita (2018)
	Miyako Island Group	Present	Fujita (2018)

## References

[B7537444] Bakhtiar E., Sartiyatno H. B (2014). The effect of time differences and bait lobster (*Panulirus* sp.) catch with krendet (trap net) fishing gear in Watukarung water, Pacitan. Journal of Fisheries Resources Utilization Management and Technology.

[B7537484] Biusing R., Chio F. L. (2004). Status of spiny lobster resources in Sabah, Malaysia. https://fishdept.sabah.gov.my/sites/default/files/uploads/file-upload/73/lobster-rooney.pdf.

[B7537435] Booth John D. (2001). Habitat preferences and behaviour of newly settled *Jasusedwardsii* (Palinuridae). Marine and Freshwater Research.

[B7549476] Bustamante R. H, Wellington G. M, Branch G. M, Edgar G. J, Martinez P, Rivera F, Smith F, Witman J, Bensted-Smith R. (2002). In a biodiversity vision for the Galapagos Islands. By Charles Darwin Foundation and World Wildlife Fund, Puerto Ayora, Galapagos..

[B7537531] Chan T. Y., Chu K. H. (1996). On the different forms of *Panuliruslongipesfemoristriga* (von Martens, 1872) (Crustacea: Decapoda: Palinuridae), with description of a new species. Journal of Natural History.

[B7537518] Chan T. Y., Carpenter K. E., Niem V. H. (1998). FAO species identification guide for fishery purposes.

[B7537540] Chan T. Y., Ng P. K. (2001). On the nomenclature of the commercially important spiny lobsters *Panuliruslongipesfemoristriga* (von Martens, 1872), *P.bispinosus* Borradaile, 1899, and *P.albiflagellum* Chan & Chu, 1996 (Decapoda, Palinuridae). Crustaceana.

[B7537549] Chen C., Fatihah S. N. (2018). A preliminary study on the distribution of spiny lobster (*Panulirus* spp.) in Labuan Island, Malaysia.. Borneo Journal of Marine Science and Aquaculture.

[B7537558] Chittleborough R., Thomas L. (1969). Larval ecology of the western Australian marine crayfish, with notes upon other palunurid larvae from the Easter Indian Ocean. Australian Journal of Marine and Freshwater Research.

[B7537582] Chow Seinen, Suzuki Nobuaki, Imai Hideyuki, Yoshimura Taku (2006). Molecular species identification of spiny lobster phyllosoma larvae of the genus *Panulirus* from the Northwestern Pacific. Marine Biotechnology.

[B7537567] Chow Seinen, Jeffs Andrew, Miyake Yoichi, Konishi Kooichi, Okazaki Makoto, Suzuki Nobuaki, Abdullah Muhamad F., Imai Hideyuki, Wakabayasi Toshie, Sakai Mitsuo (2011). Genetic isolation between the Western and Eastern Pacific Populations of Pronghorn Spiny Lobster *Panuliruspenicillatus*. PLOS One.

[B7537591] Coutures Emmanuel (2000). Distribution of phyllosoma larvae of Scyllaridae and Palinuridae (Decapoda: Palinuridea) in the south-western lagoon of New Caledonia. Marine and Freshwater Research.

[B7537600] Curtiss Anthony (1938). A short zoology of Tahiti in the Society islands.

[B7537608] Darriba Diego, Taboada Guillermo L, Doallo Ramón, Posada David (2012). jModelTest 2: more models, new heuristics and parallel computing. Nature Methods.

[B7537626] Dennis Darren M., Pitcher C. Roland, Skewes Timothy D. (2001). Distribution and transport pathways of *Panulirusornatus* (Fabricius, 1776) and *Panulirus* spp. larvae in the Coral Sea, Australia. Marine and Freshwater Research.

[B7537644] Folmer O, Black M, Hoeh W, Lutz R, Vrijenhoek R (1994). DNA primers for amplification of mitochondrial Cytochrome C Oxidase Subunit I from diverse metazoan invertebrates.. Molecular Marine Biology and Biotechnology.

[B7537654] Fujita Y. (2018). Records of decapod crustaceans in Miyako Island Group, southern Ryukyu Islands, Japan. Bulletin of the Miyakojima City Museum.

[B7537663] George R. (1965). A revision of the Indo-West Pacific spiny lobsters of the *Panulirusjaponicus* group. Deep Sea Research and Oceanographic Abstracts.

[B7537681] George R., Munro J. L. (1968). Spiny Lobsters.

[B7537694] George R. (1972). South Pacific Islands - rock lobster resources..

[B7617405] Govender Ashrenee, Groeneveld Johan, Singh Sohana, Willows-Munro Sandi (2019). The design and testing of mini-barcode markers in marine lobsters. PLOS ONE.

[B7537702] Hanis N., Siow R. (2019). Report lobster resources and fisheries in Sedili, Johor. http://repository.seafdec.or.th/bitstream/handle/20.500.12067/1504/MY-REP2019Q1_16_SP_Paper%20Lobster%20Resource%20and%20Fisheries%20In%20Sedili-SEDILI%20JOHOR_Final-sp.pdf?sequence=5&isAllowed=y.

[B7537719] Holthuis L. B. (1991). Marine Lobsters of the World: an annotated and illustrated catalogue of species of interest to fisheries known to date.

[B7537727] Ikhwanuddin M., Fatihah S. N., Nurul J. R., Zakaria M. Z. (2014). Biological features of mud spiny lobster, *Panuliruspolyphagus* (Herbst, 1793) from Johor Coastal Water of Malaysia. World Applied Sciences Journal.

[B7537808] Juinio-Menez M., Gotanco R., Williams C. K (2004). ACIAR Proceedings.

[B7538458] Juinio-Meñez Marie Antonette, Ravago Rachel (2003). Phylogenetic position of the striped-legged forms of *Panuliruslongipes* (A. Milne-Edwards, 1868) (Decapoda, Palinuridae) inferred from mitochondrial DNA sequences. Crustaceana.

[B7537821] Kalih L. (2012). Diversity and distribution of Palinurid and Scyllarid Lobster in the Lombok Island Coastal Waters.

[B7617450] Konishi Kooichi, Yanagimoto Takashi, Chow Seinen (2019). Mid- to late stage phyllosoma larvae of *Panulirusbrunneiflagellum* Sekiguchi & George, 2005 collected south of the Ogasawara Islands, Japan. Aquatic Animals.

[B7537829] Kumar Sudhir, Stecher Glen, Li Michael, Knyaz Christina, Tamura Koichiro (2018). MEGA X: molecular evolutionary genetics analysis across computing platforms. Molecular Biology and Evolution.

[B7537839] Lipcius Romuald N., Stockhausen William T., Eggleston David B. (2001). Marine reserves for Caribbean spiny lobster: empirical evaluation and theoretical metapopulation recruitment dynamics. Marine and Freshwater Research.

[B7537848] Macaranas J. M., Pante M. J. R, Juinio A. R., Gomez E. D. (unpublished data). Biochemical taxonomy of spiny lobsters and its concordance with morphological classification..

[B7537856] Matsuda H Irokazu, Yamakawa T AkashiAMAKAWA, T AKASHI (2000). The complete development and morphological changes of larval *Panuliruslongipes* (Decapoda, Palinuridae) under laboratory conditions. Fisheries Science.

[B7537865] McWilliam PS, Phillips BF (1983). Phyllosoma larvae and other crustacean macrozooplankton associated with eddy J, a warm-core eddy of south-eastern Australia. Marine and Freshwater Research.

[B7538381] Ng P. K., Naruse T. (2014). The lobsters of Christmas Island and Cocos (Keeling) Islands, with new records of *Palinurelluswieneckii* (De Man, 1881) and *Enoplometopusvoigtmanni* Türkay, 1989 (Crustacea: Decapoda: Palinuridae, Scyllaridae, Enoplometopidae). Raffles Bulletin of Zoology.

[B7538372] Ng Peter K. L, Eldredge Lucius G., Evenhuis Neal L. (2011). The names of decapod and stomatopod Crustacea from Tahiti, French Polynesia, established by Anthony Curtiss in 1938 and 1944. Zootaxa.

[B7538390] Nurfiarini Amula, Purnamaningtyas Sri Endah (2017). Pencacatan kedua dan beberapa aspek biologi lobster batik merah (*Panuliruslongipesfemoristriga* Von Martens, 1872) yang ditangkap di Teluk Sepi, Lombok barat. Jurnal Penelitian Perikanan Indonesia.

[B7538399] O'Malley Joseph M, Walsh William A (2013). Annual and long-term movement patterns of spiny lobster, *Panulirusmarginatus*, and slipper lobster, *Scyllaridessquammosus*, in the Northwestern Hawaiian Islands. Bulletin of Marine Science.

[B7618560] Palero Ferran, Guerao Guillermo, Hall Michael, Chan Tin Yam, Clark Paul F. (2014). The ‘giant phyllosoma’ are larval stages of Parribacusantarcticus (Decapoda : Scyllaridae). Invertebrate Systematics.

[B7538408] Pitcher C. R., Pitcher C. R. (1992). Spiny Lobster.

[B7538421] Poupin J., Junker M. (2008). Crustacés des îles Wallis & Futuna: inventaire illustré, espèces commercialisables et capture des formes larvaires.. https://bit.ly/3nX7HIi.

[B7538429] Ptacek Margaret B., Sarver Shane K., Childress Michael J., Herrnkind William F. (2001). Molecular phylogeny of the spiny lobster genus *Panulirus* (Decapoda: Palinuridae). Marine and Freshwater Research.

[B7549732] Radhakrishnan E. V., Kizhakudan J. K, Lakshmi Pillai S, Jeena N. S, Radhakrishnan E. V., Phillips B. F, Achamveetil G (2019). Lobsters: Biology, fisheries and aquaculture.

[B7538467] Richards Andrew H., Bell Lui J., Bell Johann D. (1994). Inshore fisheries resources of Solomon Islands. Marine Pollution Bulletin.

[B7538485] Richer de Forges B., Laboute P, Richer de Forge B. (1995). Les fonds meubles des lagons de Nouvelle-Calédonie (Sédimentologie, benthos)..

[B7538512] Sekiguchi H. (1997). Larval recruitment processes of Japanese spiny lobsters: A hypothesis.. Bulletin of Marine Science.

[B7538530] Sekiguchi Hideo, Inoue Nariaki (2002). Recent advances in larval recruitment processes of scyllarid and palinurid lobsters in Japanese waters.. Journal of Oceanography.

[B7538521] Sekiguchi Hideo, George Ray W. (2005). Description of *Panulirusbrunneiflagellum* new species with notes on its biology, evolution, and fisheries. New Zealand Journal of Marine and Freshwater Research.

[B7538539] Thompson Julie D., Gibson Toby. J., Higgins Des G. (2002). Multiple Sequence Alignment Using ClustalW and ClustalX. Current Protocols in Bioinformatics.

[B7618541] Ueda K, Yanagimoto T, Chow S, Kuroki M, Yamakawa T (2021). Molecular identification of mid to final stage slipper lobster phyllosoma larvae of the genus *Chelarctus* (Crustacea: Decapoda: Scyllaridae) collected in the Pacific with descriptions of their larval morphology. Zoological Studies.

[B7538562] Von Martens E. (1872). Ueber cubanische Crustaceen nach den Sammlungen Dr.. J. Gundlach’s. Arch Naturgesch.

[B7538581] Wahyudin Rudi Alek, Hakim A. A., Boer M., Farajallah A., Wardiatno Y. (2016). New records of *Panulirusfemoristriga* Von Martens, 1872 (Crustacea
Achelata
Palinuridae) from Celebes and Seram Islands, Indonesia. Biodiversity Journal.

[B7538571] Wahyudin Rudi A, Wardiatno Yusli, Boer Mennofatria, Farajallah Achmad, Hakim Agus A. (2017). Short Communication: A new distribution record of the mud-spiny lobster, *Panuliruspolyphagus* (Herbst, 1793) (Crustacea, Achelata, Palinuridae) in Mayalibit Bay, West Papua, Indonesia. Biodiversitas Journal of Biological Diversity.

[B7538591] Wijaya Danu, Nurfiarini Amula, Nastiti Adriani Sri, Riswanto Riswanto (2017). Kebiasaan makanan, luas dan tumpang tindih relung beberapa jenis lobster di Teluk Prigi, Kabupaten Trenggalek. BAWAL Widya Riset Perikanan Tangkap.

[B7538600] Yamauchi Mitsugu M., Miya Masaki U., Nishida Mutsumi (2002). Complete mitochondrial DNA sequence of the Japanese spiny lobster, *Panulirusjaponicus* (Crustacea: Decapoda). Gene.

[B7538609] Yeo D. C. J., Carlton J. T., Teo S. L. M., Ng P. K. L. (2011). In the wrong place - alien marine crustaceans: distribution, biology and impacts.

